# Bacterial Proliferation: Keep Dividing and Don't Mind the Gap

**DOI:** 10.1371/journal.pgen.1005757

**Published:** 2015-12-29

**Authors:** Luisa Laureti, Julien Demol, Robert P. Fuchs, Vincent Pagès

**Affiliations:** Cancer Research Center of Marseille, Team DNA Damage Tolerance, CNRS, UMR7258; Inserm, U1068; Institut Paoli-Calmettes; Aix-Marseille University, Marseille, France; University of Michigan, UNITED STATES

## Abstract

DNA Damage Tolerance (DDT) mechanisms help dealing with unrepaired DNA lesions that block replication and challenge genome integrity. Previous *in vitro* studies showed that the bacterial replicase is able to re-prime downstream of a DNA lesion, leaving behind a single-stranded DNA gap. The question remains of what happens to this gap *in vivo*. Following the insertion of a single lesion in the chromosome of a living cell, we showed that this gap is mostly filled in by Homology Directed Gap Repair in a RecA dependent manner. When cells fail to repair this gap, or when homologous recombination is impaired, cells are still able to divide, leading to the loss of the damaged chromatid, suggesting that bacteria lack a stringent cell division checkpoint mechanism. Hence, at the expense of losing one chromatid, cell survival and proliferation are ensured.

## Author Summary

DNA Damage Tolerance (DDT) mechanisms help dealing with unrepaired DNA lesions that block replication, thus challenging genome integrity. Two DDT mechanisms have previously been described: error prone Translesion Synthesis operated by specialized DNA polymerases and error free bypass that uses the information of the sister chromatid to bypass the lesion. In this work, we set up a novel genetic system that allows to insert a single DNA blocking lesion in the chromosome of a living cell and to visualize the exchange of genetic information between the undamaged and the damaged strand. Using this system, we showed *in vivo* that the replication fork is able to re-prime downstream of the lesion, leaving a gap. This gap is mostly filled in by the error free pathway through the RecA homologous recombination mechanism. We show that when the gap is left unrepaired, cells are still able to divide by losing the damaged chromatid, which evidences the lack of a stringent cell division checkpoint system.

## Introduction

All living organisms need to preserve their genetic information and to faithfully replicate their genome even in the presence of DNA damage. Despite the existence of numerous DNA repair pathways, some lesions escape repair and challenge the replication machinery by slowing down or stalling the replication fork, eventually leading to genome instability, aging and cancer. Hence, cells have developed DNA Damage Tolerance (DDT) mechanisms in order to bypass these “roadblocks” and complete replication. Two major pathways have been identified and described both in prokaryotes and eukaryotes: 1) Translesion Synthesis (TLS), which employs specialized DNA polymerases able to replicate damaged DNA, with the potential to introduce mutations (reviewed in [1]); 2) Damage avoidance pathways (also named template switching or copy choice), which use the information of the sister chromatid to bypass the lesion in a non-mutagenic way (reviewed in [2,3]). The dynamic of a replication fork encountering a DNA-blocking lesion and the processing of such lesion by Damage avoidance pathways is still poorly understood at the molecular level. In prokaryotes two models have been proposed: the first, based on the work of Rupp and Howard-Flanders [4], suggests re-priming of the replication fork behind the lesion to proceed with DNA replication, and subsequent template switching of the blocked 3’-end via invasion into the intact sister duplex promoted by the homologous recombination machinery to fill in the gap left behind. We will refer to this mechanism as "Homology Directed Gap Repair" (HDGR). The second model proposes that the bypass of the lesion occurs at the fork, where replication has stopped, and resumption of the stalled fork occurs through homologous recombination or replication fork reversal mechanisms [5–9]. In both models, TLS can also participate in filling the gap behind the fork or resuming the replication at the lesion site. Previous studies (reviewed in [10,11]) support the first model by showing that following the encounter of the replication fork with a lesion, re-priming can occur downstream of the lesion, even in the leading strand, precluding fork stalling or collapse and allowing the replication fork to move forward. These data suggest that the gaps formed downstream of lesion sites are repaired post-replicatively by Homology Directed Gap Repair pathway, as initially proposed by Rupp and Howard-Flanders [4].

Previous *in vivo* attempts to study Damage avoidance pathways in *Escherichia coli* employ lesion-containing plasmids [12–14]. However, these substrates were recently shown to be inappropriate tools, as replication fork uncoupling at lesion sites leads to full separation of the daughter strands in plasmids [15–17]. Indeed, sister chromatid cohesion is instrumental for the recombination-based transfer of information that characterizes Damage Avoidance: the loss of cohesion between sister chromatids in plasmid precludes damage avoidance to be studied in such systems. To overcome the limitation of the plasmid systems, we described in a previous work, a novel approach by which we introduced a single blocking-lesion in a specific locus of the chromosome of *E*. *coli*. We showed that under physiological conditions, error free pathways massively outweigh TLS events [17,18]. Following inactivation of the bacterial recombinase RecA, the main actor for homologous recombination mechanisms, cell viability was greatly affected by a single lesion, yet ~50% of the cells were still able to survive via unknown mechanisms, suggesting that cells are able to cope with unrepaired gaps. In the present paper, we address the question of the fate of these gaps *in vivo*: how do they impact cell proliferation? What happens to these gaps when homologous recombination mechanisms are partially impaired or abolished? In order to answer these questions, we have modified our experimental approach to directly monitor sister chromatid strand exchange in the vicinity of the lesion site.

Our new system confirms that in a *recA*+ strain, over 80% of DDT events result from a genuine sister chromatid strand exchange reaction (*i*.*e*. HDGR). Given that RecA is the major player in any strand exchange reaction, we expected a dramatic decrease in colony-forming efficiency in a *recA-* strain upon introduction of a single lesion. However, in a *recA-* strain, we only observe a two-fold reduction in colony-forming efficiency, as previously described [17]. In the present paper, we show that less than 10% of survivors in the *recA-* strain result from a genuine strand exchange reaction while the vast majority of survivors (90%) contains only the genetic markers from the undamaged strand; consequently, we refer to these colonies as resulting from "damaged chromatid loss" events. Re-priming of the replication fork behind the lesion allows the cell to pursue the replication of its chromosome, leaving a gap that might fail to be repaired or that will never be repaired as in a *recA-* strain. The lack of a stringent cell division checkpoint in bacteria allows the cell to divide despite this unrepaired gap, giving rise to a viable cell that stems from the replication of the undamaged sister chromatid only.

## Results

### A genetic tool to monitor Homology Directed Gap Repair mechanism

**Fig 1:**
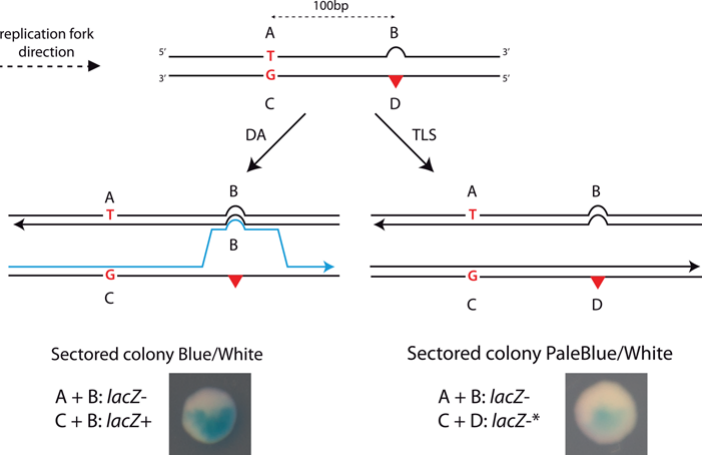
Genetic system to monitor HDGR in the presence of different DNA lesions. The system is a modified version of a previous construction used to specifically monitor TLS events [17]. The scheme represents the situation in which the lesion (red triangle) is located in the 5'-end of the *lacZ* gene in the leading orientation. The damaged strand containing the marker D, where the lesion is located, and the marker C, placed 100 bp upstream the lesion, contains a +2 frameshift in order to inactivate the *lacZ* gene. Opposite the marker D we introduced a +4 loop (marker B) that restores the reading frame of *lacZ*, and in the same strand we added marker A that contains a stop codon. Therefore the two strands are *lacZ-*. Only a mechanism of HDGR by which replication has been initiated on the damaged strand (incorporation of marker C), and where a template switch occurred at the lesion site (leading to incorporation of marker B) will restore a *lacZ*+ gene (the combination of markers C and B contains neither a stop codon nor a frameshift). *For the combination of marker C and D, we observed a leaky activity of the β-Galactosidase (due to a translational frameshift) giving rise to pale blue colonies.

Whether occurring at the fork or behind the fork, Homology Directed Gap Repair implies the exchange of genetic information between damaged and non-damaged sister chromatids. In order to monitor HDGR events (*i*.*e*. sister-strand exchange events), we designed a genetic tool illustrated in Fig 1: we constructed a vector containing a single lesion located in the coding sequence of the *lacZ* gene.

We introduced in this vector four genetic markers in order to distinguish the replication of the non-damaged strand (containing markers A and B) from the damaged strand (containing markers C and D), as well as any exchange of genetic material between the two strands. Using a combination of frameshift and stop codon, we inactivated *lacZ* gene on both the damaged (C-D) and undamaged (A-B) strand of the vector. Only a strand exchange mechanism (*i*.*e*. HDGR) by which replication has been initiated on the damaged strand (incorporation of marker C), and where a template switch occurred at the lesion site (leading to incorporation of marker B) will restore a functional *lacZ* gene (the combination of markers C and B contains neither a stop codon nor a frameshift). Using a previously described technique [17,18] this vector is integrated at a specific site in the genome of a living *E*. *coli* cell (S1A Fig). Following plating on indicator medium the resulting colonies are sectored: HDGR events appear as blue sectors, while replication of the undamaged strand appears as white sectors. It appears that because of either a translational frameshift or the presence of an alternative start codon, TLS events (*i*.*e*. combination of markers C and D) can also be monitored in this assay since they appear as pale blue sectors (Fig 1 and S1B Fig).

We implemented three different replication-blocking lesions to monitor DDT events *in vivo*: the two major UV-induced photoproducts, the thymine-thymine pyrimidine(6–4)pyrimidone photoproduct [TT(6–4)] and the cyclobutane pyrimidine dimer (TT-CPD), as well as the chemical adduct *N*-2-acetylaminofluorene covalently bound to the C^8^ of a guanine (G-AAF). As a control, for all three lesions we used a lesion-free construct. Tolerance events (i.e. the percentage of cells able to survive with the integrated lesion) are calculated by the ratio of colonies resulting from the integration of the damaged construct *versus* the lesion-free plasmid (that represents 100% of tolerance). Since the focus of the present work is to investigate lesion tolerance mechanisms, we conducted our experiments in strains devoid of repair. We thus conducted the experiments in a parental strain where nucleotide excision repair has been inactivated (*uvrA*), to avoid excision of the lesion, as well as mismatch repair (*mutS*), to prevent corrections of the genetic markers. For experiments involving the CPD lesion, the photolyase gene (*phrB*) was additionally inactivated.

**Fig 2:**
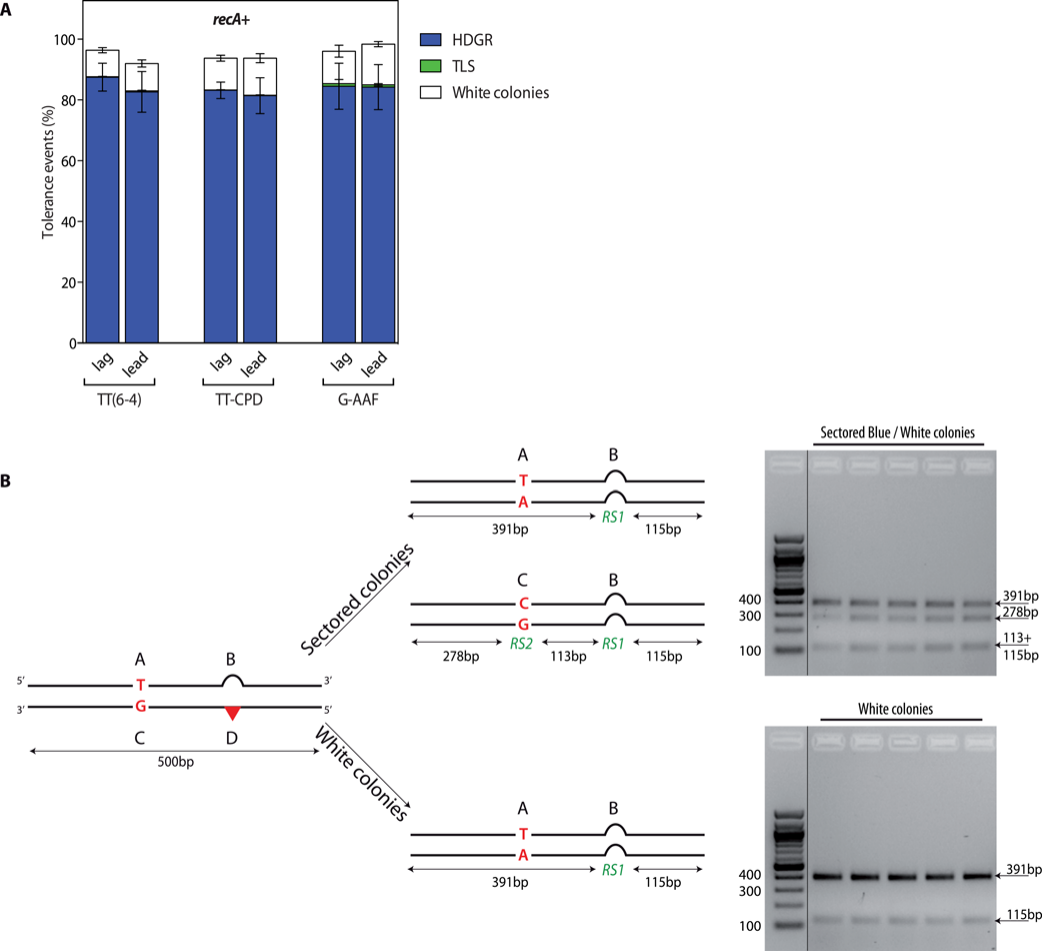
Partitioning of DDT pathways in the *recA* ^+^ strain (panel A) and molecular characterization of the colonies (panel B). A) The graph represents the partition of DDT pathways for two UV lesions, TT(6–4) and TT-CPD, and for the chemical adduct G-AAF, relative to lesion-free plasmid in a *recA* ^+^. Each lesion has been inserted in both orientations with respect to fork direction, *i*.*e*. leading (lead) and lagging (lag). Tolerance events (Y axis) represent the percentage of cells able to survive in presence of the integrated lesion compared to the lesion-free control. The data represent the average and standard deviation of at least three independent experiments. B) The digestion profile of a sectored or a white colony is represented. A 500bp PCR fragment including all four genetic markers is digested by *Ssp*I and *Pvu*II, in the case of UV lesion constructions, or by *Eco*RI and *Pvu*II, in the case of AAF lesion construction. RS1 = *Ssp*I or *Eco*RI; RS2 = *Pvu*II.

After integration of the constructs, we plated the cells before their first division so that each colony contains the progeny stemming from both strands of the mother cell. Each cell after the first replication contains a combination of the different genetic markers (A, B, C, D) that depends on the DDT mechanism that has been used (Fig 1). In our parental strain, no reduction in the colony-forming efficiency for any of the three lesions containing constructs compared to the lesion-free was observed (Fig 2A).

The majority of colonies (>80%) appeared as sectored blue/white representing HDGR events. We also measured a small contribution of TLS events as pale blue colonies (C+D markers, checked by sequencing): less than 0.3% TLS events for the UV lesions and less than 3% for the G-AAF lesion (Fig 2A), in agreement with our previous data obtained under non SOS-induced conditions [17,18]. Molecular analysis of the sectored blue/white colonies confirmed that they resulted from the processing of the lesion by HDGR, since they exhibit the expected exchange of genetic markers A+B and C+B (*i*.*e*. white and blue) (Fig 2B). Additionally, we also observed the presence of white colonies (∼10%) that were found, after molecular analysis, to contain only the genetic markers A+B of the undamaged strand (Fig 2A and 2B). We will discuss this class of events in the next section. The same partitioning of DDT events was obtained whether the lesion was introduced on the leading or on the lagging strand.

### DNA damage tolerance when homologous recombination is impaired

While the precise mechanism for HDGR has not yet been established, it is generally accepted that the missing genetic information near the lesion site is accurately retrieved from the sister chromatid by mechanisms akin to homologous recombination (HR). As RecA plays an important role in homologous recombinational repair mechanisms by promoting strand invasion and homology pairing [19], we wanted to measure the level of HDGR in a strain where HR is abolished (*i*.*e*. in the absence of RecA) and to monitor the impact on cell proliferation.

**Fig 3:**
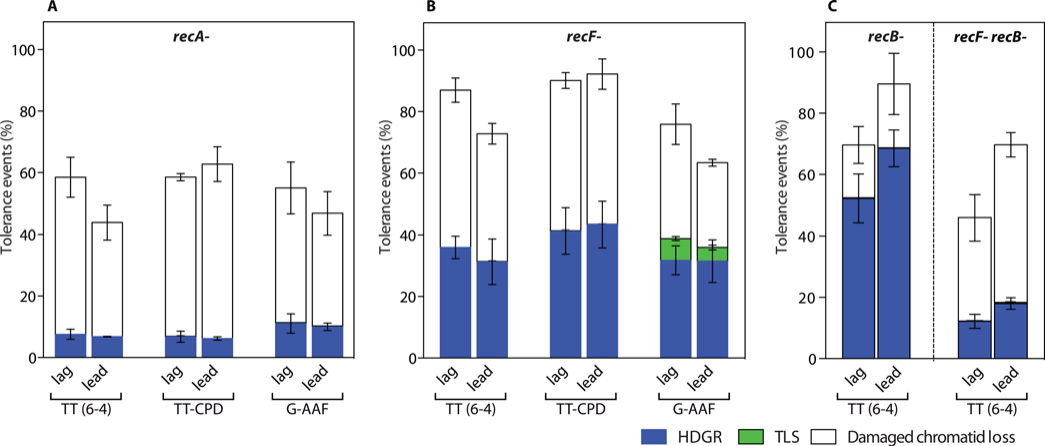
Partitioning of DDT pathways in homologous recombination impaired strains. The graph represents the partition of DDT mechanisms for two UV lesions, TT6-4 and TT-CPD, and for the chemical adduct G-AAF, relative to lesion-free plasmid, in a *recA-* strain (**A**), in a *recF-* strain (**B**), and for the TT6-4 lesion in a *recB-* and *recB-recF-* strain (**C**). Each lesion has been inserted in both orientation of the replication fork, *i*.*e*. leading (lead) and lagging (lag). Tolerance events (Y axis) represent the percentage of cells able to survive in presence of the integrated lesion compared to the lesion-free control. The data represent the average and standard deviation of at least three independent experiments.

Introducing the lesions in a *recA-* strain caused a ~50% drop of cell survival as previously shown [17]. Among the survivors, only 7–13% of tolerance events appeared as blue/white sectored colonies representative of strand exchange events (Fig 3A).

These data show that RecA is strongly involved in HDGR mechanisms. The majority of these surviving colonies (~85%) were white and their molecular analysis showed only the presence of the genetic markers of the undamaged strand (A+B) (Figs 3A and 2B). It appears in these colonies that the genetic information of the damaged chromatid was lost as neither the markers C nor D were recovered. In order to assess whether the loss was only local or more widely spread, we generated two additional sets of constructions: i) where an additional set of markers (E and F) was located 1.6kb downstream the lesion site (with respect to the replication fork direction) (S2A Fig); ii) where the markers A and C were located further upstream of the lesion (800bp) (S2B Fig). Both constructs led to the same partitioning of DDT events as the initial construct (see Figs 2A and 3A). The molecular analysis of the white colonies showed that all markers of the damaged strand were lost, regardless of their distance to the lesion (S2A and S2B Fig). Two events could cause the loss of these markers (on a distance of at least 2.4kb) and lead to the formation of white colonies: i) the total loss of the damaged chromatid, or ii) extended resection beyond the markers by exonucleases such as RecBCD, a nuclease complex known for its voracity in degrading DNA in the presence of a double strand break [20], or by other 3’→5’ exonucleases. If such a resection occurred, RecA-dependent HR repair could take place, generating a *lacZ-* colony, which contains only the markers A+B. In order to test this hypothesis, we measured DDT pathways in the presence of the TT(6–4) lesion in a *recB*- strain (Fig 3C): while we previously observed ~10% of white colonies in the parental strain, in the absence of the *recB* gene this number didn't decrease, as we would expect if the white colonies were the results of extended strand resection. These data clearly indicate that strand resection, at least by RecBCD nuclease doesn't account for the white colonies we observe. Hence, our data suggest the loss of the entire damaged chromatid and we named this phenomenon "damaged chromatid loss".

Even though we still observed some genuine strand exchange (around 10%) in a *recA-* strain, damaged chromatid loss accounts for the majority (~85%) of DDT events when homologous recombination is abolished. It appears, therefore, that damaged chromatid loss occurs infrequently (~10%) in HR-proficient strain but it prevails in a HR deficient strain. What happens in a situation where HR is not completely abolished but only impaired or delayed? In order to address this question, we monitored DDT events in a *recF* deficient strain in which HR is partially affected. *E*. *coli* possesses two post-replication repair pathways that rely on homologous recombination: the RecBCD pathway is involved in the repair of double-strand DNA breaks, while the RecF pathway participates in the repair of single-stranded DNA (ssDNA) gaps formed when the replication fork encounters DNA blocking lesions and proceeds past the damage [19, 21]. RecF is part of the RecFOR complex that mediates the loading of RecA on SSB-coated ssDNA and stabilizes the formation of the RecA nucleofilament, necessary for homology search and strand pairing [22]. Therefore, *recF* inactivation delays RecA filament formation and consequently partially impairs daughter strand gap repair. Following insertion of any of the three DNA blocking lesions in a *recF-* strain, we did not observe any strong loss of viability. However, we observed a two-fold decrease in HDGR events (sectored blue/white colonies) and a proportional increase in damaged chromatid loss events (~50%) (Fig 3B). This number is similar in the double mutant *recF-recB-* (Fig 3C), indicating that strand resection by RecBCD nuclease doesn't account for the white colonies we observe. Thus, in the absence of the recombination mediator proteins RecFOR, HDGR can still occur but with reduced efficiency. The assembly of the HDGR machinery is possibly delayed and damaged chromatid loss events take over, allowing to reach high cell survival. In this strain, we also observed a four-fold increase in -2 frameshift TLS, the signature of PolII-dependent TLS at the G-AAF lesion (Fig 3B) [23] showing that when the HDGR pathway is slightly impaired TLS events might be favored. No TLS increase was observed for the two UV lesions since they are exclusively bypassed by PolV that directly requires activation by the RecA filament [24].

Additionally, we observed in both the *recB-* and the *recF-recB-* mutants a ~20% decrease in the number of blue colonies (Fig 3C). This suggests a role for RecB in the HDGR mechanisms.

### Lesions on opposite strands cause lethality

Our results indicate that the presence of a non-repairable gap in one of the chromatids does not block cell division, but gives rise to a daughter cell originating from the undamaged chromatid. This phenomenon, which involves the loss of the damaged chromatid, should in principle lead to 100% survival (since the cells are able to generate a progeny). Experimentally, upon introduction of a single lesion in a *recA*- strain, we observed the extent of survival to be ~50% as compared to the integration of the lesion-free vector (Fig 3A). While we specifically introduced a single lesion in one of the strands, we reasoned that, in addition, the *E*. *coli* chromosome might contain additional endogenous lesions that would cause the observed loss in survival. To investigate the fate of non-repairable lesions in opposite strands in the *E*. *coli* chromosome, we constructed a plasmid that contains a single TT(6–4) lesion on one strand and an average of two to three UV lesions randomly distributed in the opposite strand. When integrated in a *recA-* strain, the presence of clustered lesions on the complementary strand turned out to be highly toxic: damaged chromatid loss, which was the main way to survive in a *recA-* strain in the presence of a single DNA replication-blocking lesion, is now completely abolished and essentially no cell survival was observed (S3 Fig). These data clearly show that a HR-deficient cell can survive despite the presence of a non-repairable gap in one chromatid only if the other chromatid can get fully replicated. In conclusion, the experimentally determined 50% survival rate in a population of *recA-* cells that contains an artificially introduced single lesion in one chromosome strand seem to suggest that on average half of the cells contain at least one additional endogenous replication-blocking lesion in the opposite strand.

## Discussion

In this study, we developed a genetic tool to monitor *in vivo* sister-strand exchange events, following the insertion of a single lesion into the chromosome of *E*. *coli*. Our system confirmed that under physiological conditions, homology directed gap repair is the major pathway cells implement to bypass blocking lesions and it proved to be dependent on the activity of the recombinase RecA mainly through the RecF pathway and to a lesser extent via RecB.

**Fig 4:**
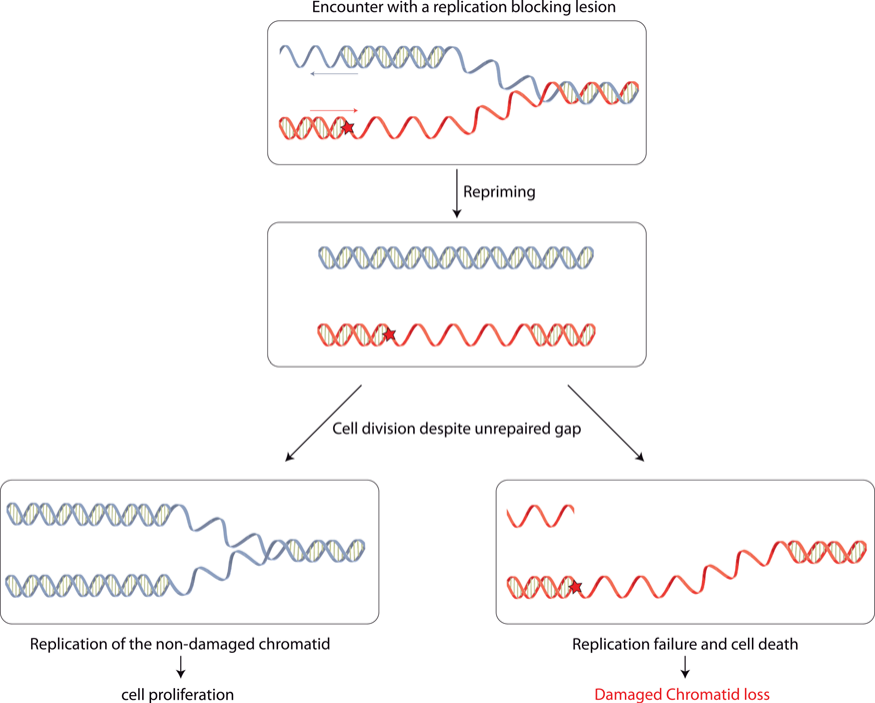
Model for damaged chromatid loss event. After encountering a DNA replication-blocking lesion, either in the leading or in the lagging strand, the replication fork is able to restart behind the lesion to resume replication. The gap left opposite the lesion will be filled in by HDGR mechanism (or by TLS). However, when HDGR fails or when homologous recombination is impaired, as in a *recF-* strain, or completely abolished, as in a *recA-* strain, the gap will not be repaired. Despite the presence of such unrepaired gap, cells keep dividing: the daughter cell that inherits the lesion and the unrepaired gap will die, while the daughter cell stemming from the replication of the undamaged chromatid will survive.

Interestingly, our present work also shows that when this mechanism of post-replication repair fails, cells are still able to survive despite the presence of a single non-repairable gap in one of their chromatids. We refer to this strategy as "damaged chromatid loss" and propose the following model (Fig 4): when encountering a replication-blocking lesion, either in the leading or in the lagging strand, fork restart downstream the lesion allows replication to proceed on both strands, leaving a gap opposite the lesion.

In a strain where the repair mechanisms are inactivated (NER-) but where all DNA damage tolerance pathways are functional, this gap is filled mainly by HDGR mechanism (∼90%) and rarely by TLS polymerases (< 2%). However, a small fraction of cells may fail to repair the gap. This situation is exacerbated in the absence of the RecA recombinase (*i*.*e*. a *recA-* strain), where the gap opposite the lesion is very inefficiently filled in by HDGR mechanism or TLS. Our data show that the presence of an unrepaired gap does not prevent colony-forming ability: the daughter cell that inherits the unrepaired chromosome (containing a gap and a lesion) will not be able to survive during the next round of replication, while the other daughter cell will give rise to a colony, containing only the genetic markers from the undamaged chromatid (Fig 4). This result implies the lack of a stringent cell division checkpoint system that would prevent cell division in the presence of a single unrepaired gap on one of the chromatids. In a *recA*+ strain, damaged chromatid loss events occur in 10% of the cells, suggesting that rapid proliferation can occasionally lead to chromatid loss even when HR mechanisms are functional. We also show that when HR is partially impaired (*i*.*e*. in a *recF*- strain), the overall colony-forming efficiency is essentially preserved (80–90%). However, compared to a *recF*+ strain, the profile of the surviving colonies is strongly modified as colonies survive equally well via genuine HDGR (~40%) and via damaged chromatid loss (~40%). When HR is completely abolished (*i*.*e*. in a *recA*- strain), survival accompanied by the loss of the damaged chromatid is the major event. Still, in this strain the overall survival is only around 50%. This loss of viability could be accounted for the presence of at least an additional endogenous lesion in the opposite strand, thus leading to a defect in replication of both chromatids. The data obtained with a few clustered lesions in the opposite strand (S3 Fig) show that no additional replication-blocking DNA lesion in the opposite strand can be tolerated. This confirms that the one condition for damaged chromatid loss to occur is the absence of simultaneous replication-blocking DNA lesions in the opposite strand that would otherwise preclude full replication of both chromatids. These results corroborate the previous observation from Howard-Flanders [25] where they estimated that an average of 1.3 lesions in the genome is lethal following UV irradiation of an *uvrA recA* double mutant. Interestingly, even in the absence of RecA, we were still able to observe 7–13% of blue/white sectored colonies that after molecular analysis showed to contain the markers A+B and C+B resulting from a strand exchange event. This observation suggests the existence of a RecA-independent recombination mechanism, as already proposed in [13,26,27]. In the future, we would like to exploit our genetic system to further characterize all the proteins involved in the RecA-independent pathway.

Our data and the proposed model that arises from it point out two essential features of the dynamic of the replication fork that encounters a lesion: i) skipping of the lesion and repriming downstream the lesion is occurring, allowing the cell to complete the replication of its chromosome; ii) the bacterial cell lacks an efficient cell division checkpoint system that would prevent its division despite the presence of an unrepaired gap. These two points are discussed below as well as their evolutionary significance.

### Discontinuous model for replication of damaged DNA

It has been controversial whether lesion tolerance occurs at the fork (continuous model) or behind the fork (discontinuous model) [10,11]. The work of Rupp and Howard-Flanders on UV-irradiated cells showed the formation of daughter strand gaps following replication fork stalling [4,28], thus suggesting that replication of a UV-damaged chromosome is discontinuous on both strands (not only on the lagging strand). The discontinuous replication model has also been proposed for *E*. *coli* under normal replication condition [29,30]. In support to the discontinuous model, more recent *in vitro* studies showed that *E*. *coli* replication fork can restart even after encountering a lesion on the leading strand and this mechanism is dependent on the DnaG primase [31–34]. The RecF recombinational pathway has been associated with the repair of daughter strand gaps [19]. We showed that in a *recF* mutant, HDGR mechanisms are partially impaired (because of a delay in the RecA filament formation and strand exchange mechanism), leading to an increase in damaged chromatid loss events that allows to maintain cell survival and proliferation up to 80–90%. Recently, Kowalczykowski’s group [35] showed *in vitro* that the RecFOR complex specifically loads RecA to ssDNA gap adjacent to a 5' end of dsDNA-ssDNA junction. Interestingly, they showed that RecF recognizes junction containing either DNA or RNA at the 5' terminus, suggesting a role in the repair of gaps both in leading and lagging strands. Our *in vivo* genetic data confirms this hypothesis: inactivation of *recF* produces the same effect on DDT mechanisms whether the blocking lesion is located on the leading or the lagging strand (Fig 3B), indicating that most likely the same mechanism of gap filling is employed for both strands.

In addition, we show that RecB also participates, although to a lesser extent, to the HDGR mechanisms. The deletion of *recB* leads to ~20% decrease in the use of HDGR both in a *recF*+ and a *recF*- background (Fig 3C). To our knowledge, only Wang and Smith [36] have proposed a role for RecB in gap repair following UV irradiation. The effect of *recB* deletion that we observe here is very unlikely to be related to the formation of double strand breaks (DSB) since the repair of DSB would lead to extended resection that would remove the markers of the damaged strand (and lead to the formation of white colonies). Instead, the marker upstream the lesion (marker C) is still present and gives rise to blue colonies, the hallmark of HDGR.

Hence, our results point to a daughter strand gap repair model involving mainly the recombinational RecF pathway and RecB to a lesser extent. These observations provide *in vivo* evidence for a re-priming mechanism in both leading and lagging strands and favor the discontinuous replication model of damaged DNA. Since uncoupling of leading and lagging strand synthesis is only possible over a short distance [31], re-priming and replication restart behind the lesion is an efficient and rapid way to complete replication of the undamaged chromatid, allowing the cell to give rise to a progeny.

### In the absence of a stringent cell division checkpoint, cells can divide despite the presence of an unrepaired gap in one chromatid

In order to achieve damaged chromatid loss, cells not only have to fully replicate the non-damaged chromatid following a re-priming event, but also they have to be able to divide despite the presence of an un-repaired gap at the lesion site. Our data show that cell division is possible even when the gap is not repaired by HDGR. This result points out to the absence of a stringent DNA damage checkpoint that would prevent cell division. *E*. *coli* has been described to possess a checkpoint-like system: it is embodied by the *sulA* (*sfiA*) gene product that acts as an inhibitor of cell division [37]. *sulA* promoter is controlled by the SOS system and is induced in response to DNA damage. However, in a situation like our experimental conditions where the level of damage is low, the threshold of ssDNA formation and SOS induction is most likely too weak to induce sufficient level of SulA protein to block cell division. The inactivation of *sulA* led to the same outcome in term of survival, HDGR and damaged chromatid loss (S4 Fig). Hence, cells are able to divide despite the presence of an unrepaired gap, leading to the loss of the damaged chromatid. Eukaryotic cells also possess several checkpoint mechanisms that are able to sense DNA damage or replication stress and delay cell division until repair is achieved (or cells might undergo apoptosis) [38–40]. However, even in eukaryotic cells, the level of checkpoint activation is directly correlated to the level and the nature of DNA damage or replication stress. A previous study in budding yeast showed that a single DNA double-strand break induced Rad9-mediated arrest and resulted in telomere loss, yet some cells are still able to recover from the arrest and propagate for some generations even in the presence of an unrepaired chromosome [41]. Very recently, Mohebi *et al* [42] showed that following replication fork arrest, replication restart and recombination intermediates are not sufficient to activate the checkpoint in the first cell cycle. It has also been proposed that the presence of gaps resulting from replication of common fragile sites does not prevent mitosis and that the gaps are filled in later during cell cycle [43]. These data, together with ours, seem to suggest that both prokaryotes and eukaryotes can cope with at least one or few unrepaired lesions or non-replicated DNA region, allowing cell division. While in eukaryotes this strategy is employed to delay the repair to a more favorable time, in prokaryotes, by dividing the cell gives rise to a living progeny stemming from the undamaged chromatid and loses the damaged chromatid allowing rapid proliferation.

In conclusion, we show *in vivo* that re-priming downstream of a replication blocking lesion can occur either on the lagging or on the leading strand, leaving a gap behind the replication fork, and that *E*. *coli* cells are able to divide despite the presence of an unrepaired gap, thanks to the absence of a stringent cell division checkpoint system. Whereas re-priming of the replication fork was shown to allow rapid DNA replication, the damaged chromatid loss strategy that we show here allows rapid proliferation. It appears that in bacteria evolution has favored cell proliferation over accurate DNA repair.

## Materials and Methods

### Bacterial strains and growth conditions

All *E*. *coli* strains used in this work are derivative of strains FBG151 and FBG152 [17,44] and were grown on solid and in liquid Lysogeny Broth (LB) medium. Gene disruptions of *recA*, *recF*, *mutS*, *uvrA*, *phrB*, *recB* and *sulA* were achieved by the one-step PCR method [45]. The following FBG151 or FBG152 derived strains were constructed by P1 transduction: EVP22 and 23 (FBG151 and FBG152 uvrA::frt mutS::frt), EVP122 and 123 (FBG151 and FBG152 uvrA::frt mutS::frt recA::frt), EVP49 and 50 (FBG151 and FBG152 uvrA::frt mutS::frt recF::cm), EVP101 and 102 (FBG 151 and FBG152 uvrA::frt mutS::frt sulA::frt), EVP164 and 165 (FBG151 and FBG152 uvrA::frt mutS::frt recB::frt), EVP576 and 577 (FBG151 and FBG152 uvrA::frt mutS::frt recF::cm recB::frt), EVP183 and 184 (FBG151 and FBG152 uvrA::frt mutS::frt phrB::frt), EVP205 and 206 (FBG151 and FBG152 uvrA::frt mutS::frt phrB::frt recA::frt) and EVP498 and 499 (FBG151 and FBG152 uvrA::frt mutS::frt phrB::frt recF::frt). All strains carry the plasmid pVP135 that allows the expression of the int–xis genes under the control of IPTG. Following the site-specific recombination reaction, the lesion [G-AAF, TT-CPD or TT(6–4)] is located either in the lagging strand (FBG151 derived strains) or in the leading strand (FBG152 derived strains). Antibiotics were used at the following concentrations: ampicillin 50 or 100 μg/ml; tetracycline 10 μg/ml and kanamycin 100 μg/ml. When necessary IPTG and X-Gal were added to the medium at 0.2mM and 80 μg/ml, respectively.

### Plasmids

pVP135 expresses the integrase and excisionase (int–xis) genes from phage lambda under the control of a trc promoter that has been weakened by mutations in the -35 and the -10 region [46]. Transcription from Ptrc is regulated by the lac repressor, supplied by a copy of lacI^q^ on the plasmid. The vector has been modified as previously described [17].

pVP146 is derived from pACYC184 plasmid where the chloramphenicol resistance gene has been deleted by *Bsa*AI digestion and re-ligation. This vector, which carries only the tetracycline resistance gene, serves as an internal control for transformation efficiency.

pLL1, pLL2c, pLL4, pLL5, pLL7 and pLL9 are derived from pVP141 [17] and contain several genetic markers as described in the result section. These plasmid vectors contain the following characteristics: the ampicillin resistance gene, the R6K replication origin that allows plasmid replication only if the recipient strain carries the pir gene [47], and the 5’ end of the *lacZ* gene in fusion with the attL site-specific recombination site of phage lambda. The P’3 site of attL has been mutated (AATCATTAT to AATTATTAT) to avoid the excision of the plasmid once integrated [48]. All plasmids are produced in strain EC100D pir-116 (from Epicentre Biotechnologies, cat# EC6P0950H) in which the pir-116 allele supports higher copy number of R6K origin plasmids. Vectors carrying a single lesion for integration were constructed as described previously [17] following the gap-duplex method [49]. A 13-mer oligonucleotide, 5′-GCAAGTTAACACG-3′, containing no lesion, a TT-CPD or a TT(6–4) lesion (underlined) in the *Hinc*II site was inserted into the gapped-duplex pLL1/2c, pLL4/5 or pLL9/2c, leading to an out of frame *lacZ* gene. A 15-mer oligonucleotide 5′-ATCACCGGCGCCACA-3′ containing or not a single G-AAF adduct (underlined) in the *Nar*I site was inserted into the gapped-duplex pLL1/7.

For the construction with multiple UV lesions, before the preparation of the gapped-duplex pLL1/2c, the linearized plasmid pLL2c was irradiated with a high dose of UV (200J/m^2^) in order to contain 2–3 UV lesions on each strand.

### Monitoring HDGR

To 40 μL aliquot of competent cells, prepared as previously described [17], 1 ng of the lesion-free control plasmid, or 1 ng of the lesion-carrying vector mixed with 1 ng of the internal standard (pVP146) was added and electroporated in a GenePulser Xcell from BioRad (2.5 kV, 25 μF, 200 Ω). Cells were first resuspended in super optimal broth with catabolic repressor (SOC), then diluted in LB containing 0,2 mM IPTG. Cells were incubated for 45 min at 37°C. Part of the cells were plated on LB + 10 μg/mL tetracycline to measure the transformation efficiency of plasmid pVP146, and the rest were plated on LB + 50 μg/mL ampicillin + 80 μg/mL X-gal to select for integrants (Amp^R^) and to visualize HDGR events (*lacZ+* phenotype). Cells were diluted and plated using the automatic serial diluter and plater EasySpiral Dilute (Interscience). Colonies were counted using the Scan 1200 automatic colony counter (Interscience). The integration rate is about 2,000 clones per picogram of vector for our parental strain.

We plated before the first cell division; therefore, following the integration of the damaged vector, sectored blue/white colonies represent HDGR events; sectored pale blue/white colonies represent TLS events and pure white colonies represent damaged chromatid loss event. The relative integration efficiencies of lesion-carrying vectors compared with their lesion-free homologues, and normalized by the transformation efficiency of pVP146 plasmid in the same electroporation experiment, allow the overall rate of lesion tolerance to be measured. Even in the absence of the mismatch repair system, we observed a small percentage (∼5%) of correction of the markers B/D that we took into account for the interpretation of our results.

For the plasmid constructions containing clustered UV lesions (pLL1/2c TT0/UV and pLL1/2c TT6-4/UV), we corrected the survival data taking into account the percentage of plasmids that after UV irradiation do not have any DNA lesions. The number of lesion-free plasmids follows a Poisson distribution law and in our case it is equal to 7.73%.

### Molecular analysis

An average of 80 clones (sectored blue/white or white) was analysed for each construction in every strain (*i*.*e*. the parental strain, *recA* and *recF* strain). For the constructions pLL1/2c and pLL1/7, to amplify the sequence containing the four markers (A, B, C, D), the couple of primers VP56 (TAAATGTGAGCGAGTAACAACC) and VP215 (CTTGGGCTGCAGGTCGACT) was used. The PCR products of 506 bp were then digested by *Ssp*I and *Pvu*II, for the UV lesions, and by *Eco*RI and *Pvu*II, for the G-AAF lesion. The digestion profile was analysed by electrophoresis on a 1.8% of agarose gel. For the construction pLL4/5, to amplify the sequence containing the four markers (A, B, C, D), the couple of primers VP210 (TCGGGTTTTCGACGTTCAGA) and VP215 (CTTGGGCTGCAGGTCGACT) was used. The PCR products of 1100 bp were then digested by *Ssp*I and *Nhe*I and analysed on agarose gel. For the construction pLL9/2c, to amplify the sequence containing the six markers (A, B, C, D, E, F) the primers VP56 and GM1 (GCGCTAATGCTCTGTTACAGG) were used. The PCR products of 2283 bp were digested by *Bgl*II and *Ssp*I and analysed on agarose gel.

## Supporting Information

S1 Fig Integration of a single lesion into the chromosome (panel A) and molecular analysis of colonies sectors (panel B). **A)** The recipient strain contains a single attR integration site in fusion with the 3' end of *lacZ* gene at min 17 in the *E*. *coli* chromosome. Following ectopic expression of phage lambda integrase and excisionase, the lesion-carrying construct is introduced by electroporation. Its attL site will recombine with the chromosomal attR, leading to integration of the entire lesion-containing construct. Integration events are selected on the basis of their resistance to ampicillin. The exchange of genetics markers between the damaged and the non-damaged strand (HDGR events) restores a functional *lacZ* gene leading to the formation of blue sectors on X-gal indicator plates. **B)** Molecular analysis (by restriction and sequencing) of the different sectors confirmed that dark blue sectors are the results of HDGR mechanisms (C+B markers), white sectors result from damaged chromatid loss (A+B markers) and pale blue sectors result from TLS events (C+D markers).
(PDF)

S2 Fig Design of the genetic constructions pLL9/2c (panel A) and pLL4/5 (panel B). **A)** We modified the first construction (Fig 1) by creating another mismatch (C/T) 1.6 Kb downstream the 5'-end of the *lacZ* gene (with respect to the replication fork direction). The new genetic markers are indicated with the letters E and F. If a damaged chromatid loss event occurs, only white colonies containing the marker A+B+E would be observed. The molecular analysis of the white colonies confirmed our hypothesis. **B)** We modified the first construction (Fig 1) by moving the genetic markers A/C 800 bp upstream the lesion (with respect to the replication fork direction). As observed in the previous constructions, all the white colonies contain the genetic markers A+B, as result of a damaged chromatid loss event.
(PDF)

S3 Fig Clustered lesions on opposite strands inhibit damaged chromatid loss. The graph represents the partition of DDT mechanisms (HDGR, TLS and damaged chromatid loss) after integration of the plasmid containing a single TT6-4 lesion (indicated as TT6-4) and a plasmid containing a single TT6-4 lesion and an average of 2–3 UV lesions in the complementary strand (indicated as TT6-4/UV) in a *recA* deficient strain. The TT6-4 lesion has been inserted in both orientation of the replication fort, *i*.*e*. leading (lead) and lagging (lag). Tolerance events (Y axis) represent the percentage of cells able to survive in presence of the integrated lesion compared to the lesion-free control. The data represent the average and standard deviation of at least three independent experiments. The data for the construction TT6-4/UV have been corrected taking into account the percentage of plasmids without additional UV lesions in the complementary strand (see Methods). ND = no cell survival was observed.
(PDF)

S4 Fig Partitioning of DDT pathways in a *sulA* deficient strain. The graph represents the partition of DDT mechanisms for the UV lesion TT6-4 relative to lesion-free plasmid, in a *sulA-* strain. The lesion has been inserted in both orientation of the replication fork, *i*.*e*. leading (lead) and lagging (lag). Tolerance events (Y axis) represent the percentage of cells able to survive in presence of the integrated lesion compared to the lesion-free control. The data represent the average and standard deviation of at least three independent experiments. For a better comparison we included in the graph the previous results obtained in the parental strain.
(PDF)
