# Supplementary figures and images for: Bacterial Proliferation: Keep Dividing and Don't Mind the Gap
Source: PLoS Genet. 2015 Dec 29;11(12):e1005757. doi: 10.1371/journal.pgen.1005757 (PMC4699847; doi:10.1371/journal.pgen.1005757)

S1 Fig

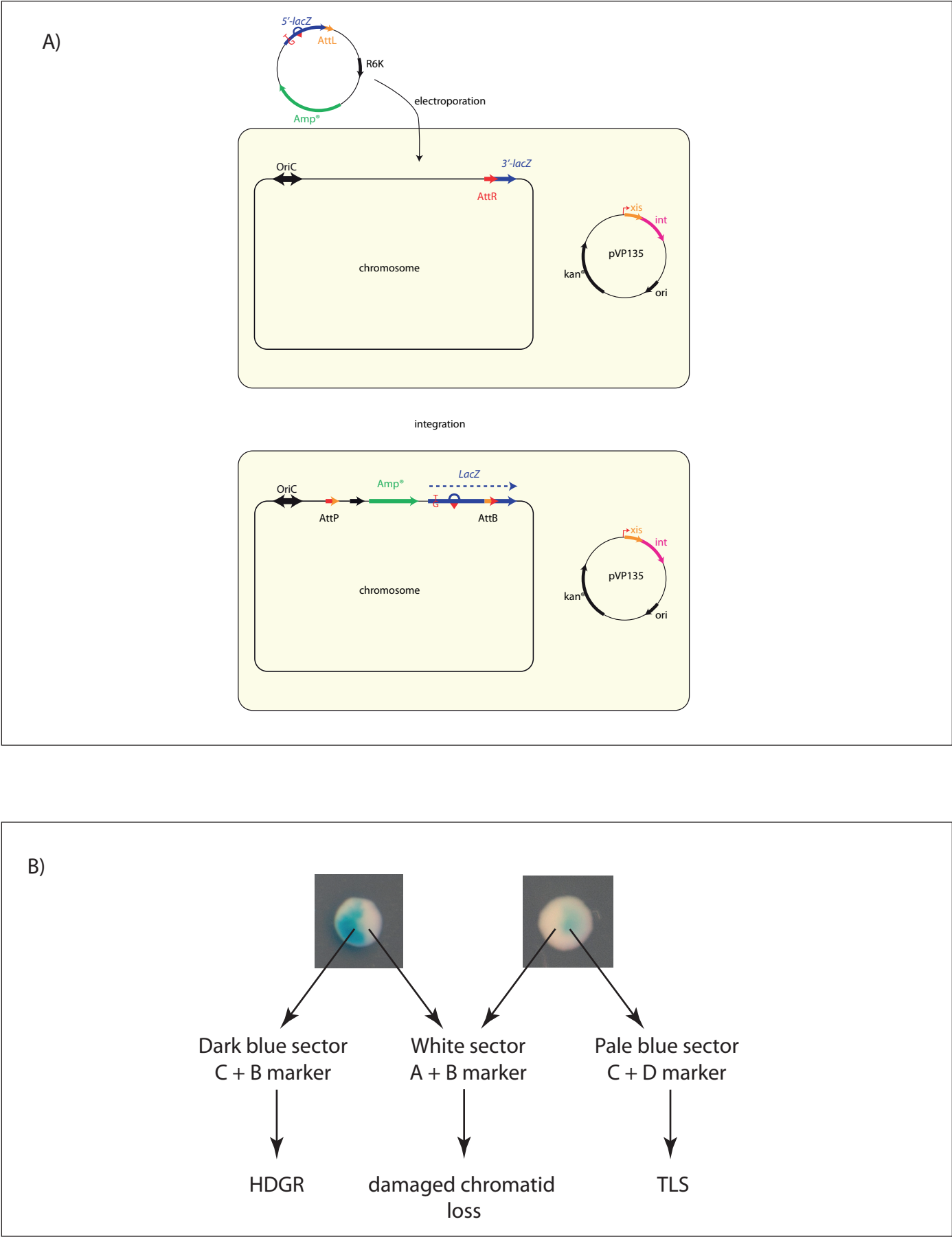

Supplement: S1 Fig — A) The recipient strain contains a single attR integration site in fusion with the 3' end of lacZ gene at min 17 in the E. coli chromosome. Following ectopic expression of phage lambda integrase and excisionase, the lesion-carrying construct is introduced by electroporation. Its attL site will recombine with the chromosomal attR, leading to integration of the entire lesion-containing construct. Integration events are selected on the basis of their resistance to ampicillin. The exchange of genetics markers between the damaged and the non-damaged strand (HDGR events) restores a functional lacZ gene leading to the formation of blue sectors on X-gal indicator plates. B) Molecular analysis (by restriction and sequencing) of the different sectors confirmed that dark blue sectors are the results of HDGR mechanisms (C+B markers), white sectors result from damaged chromatid loss (A+B markers) and pale blue sectors result from TLS events (C+D markers). (PDF) [file pgen.1005757.s001.pdf]

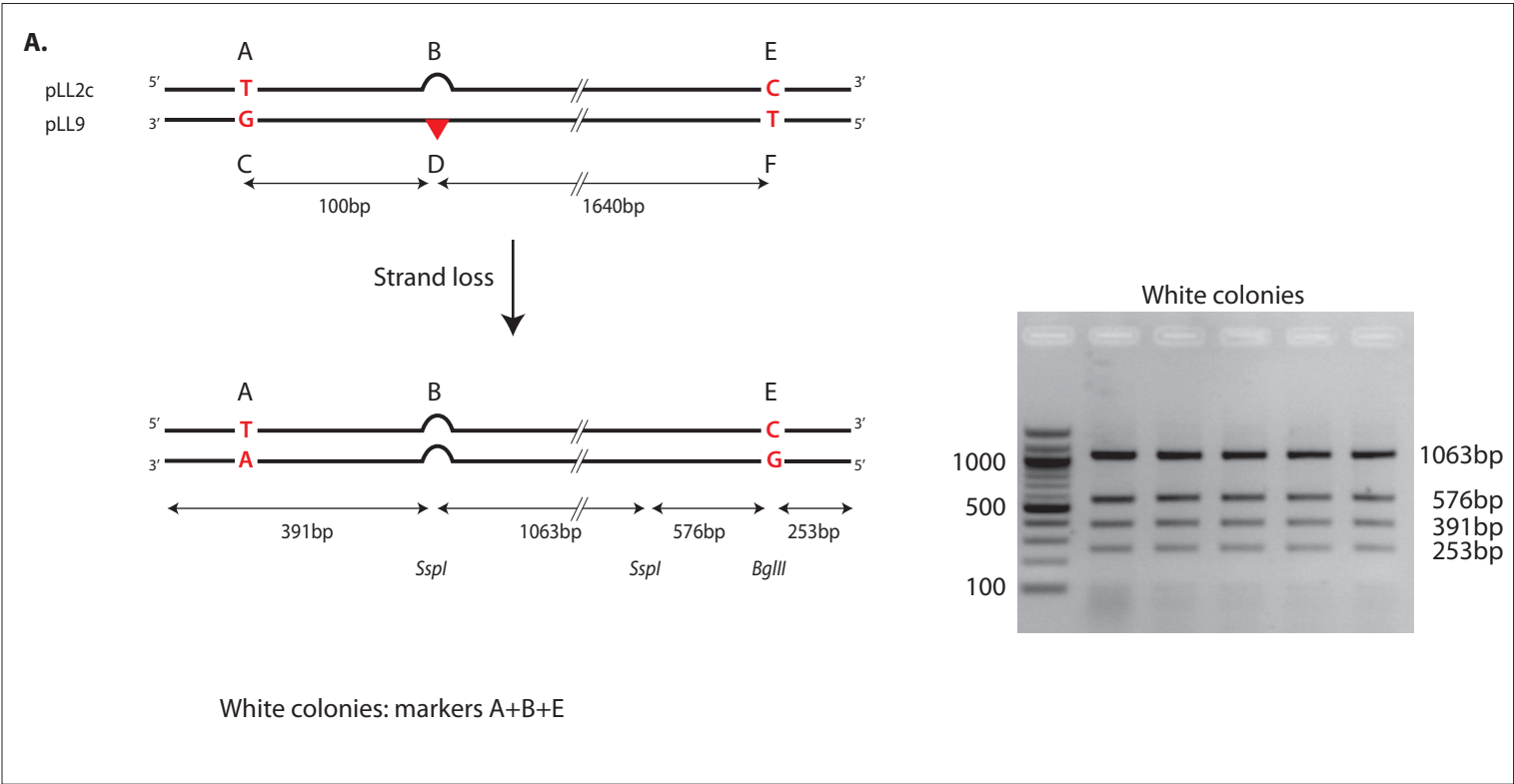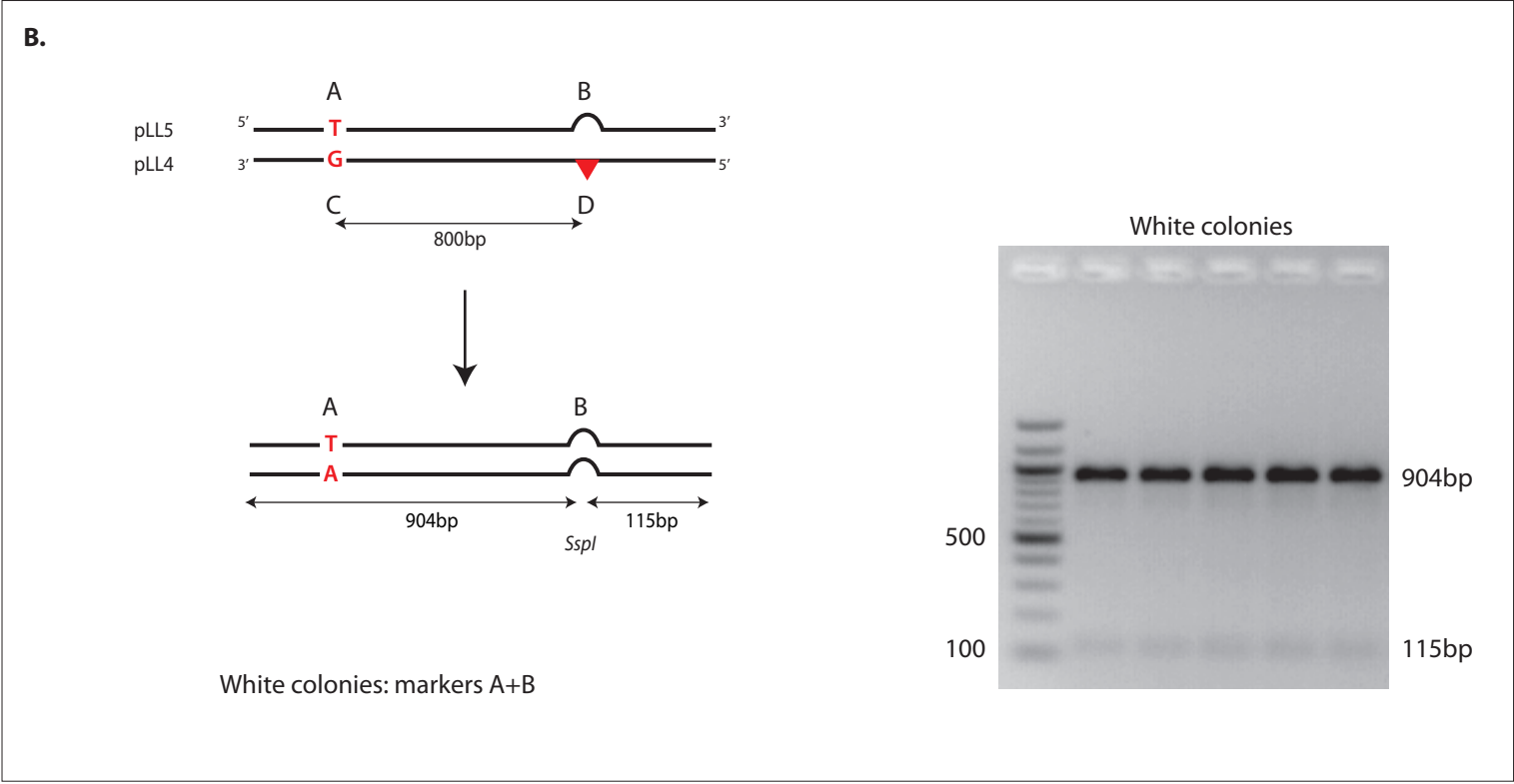

Supplement: S2 Fig — A) We modified the first construction (Fig 1) by creating another mismatch (C/T) 1.6 Kb downstream the 5'-end of the lacZ gene (with respect to the replication fork direction). The new genetic markers are indicated with the letters E and F. If a damaged chromatid loss event occurs, only white colonies containing the marker A+B+E would be observed. The molecular analysis of the white colonies confirmed our hypothesis. B) We modified the first construction (Fig 1) by moving the genetic markers A/C 800 bp upstream the lesion (with respect to the replication fork direction). As observed in the previous constructions, all the white colonies contain the genetic markers A+B, as result of a damaged chromatid loss event. (PDF) [file pgen.1005757.s002.pdf]

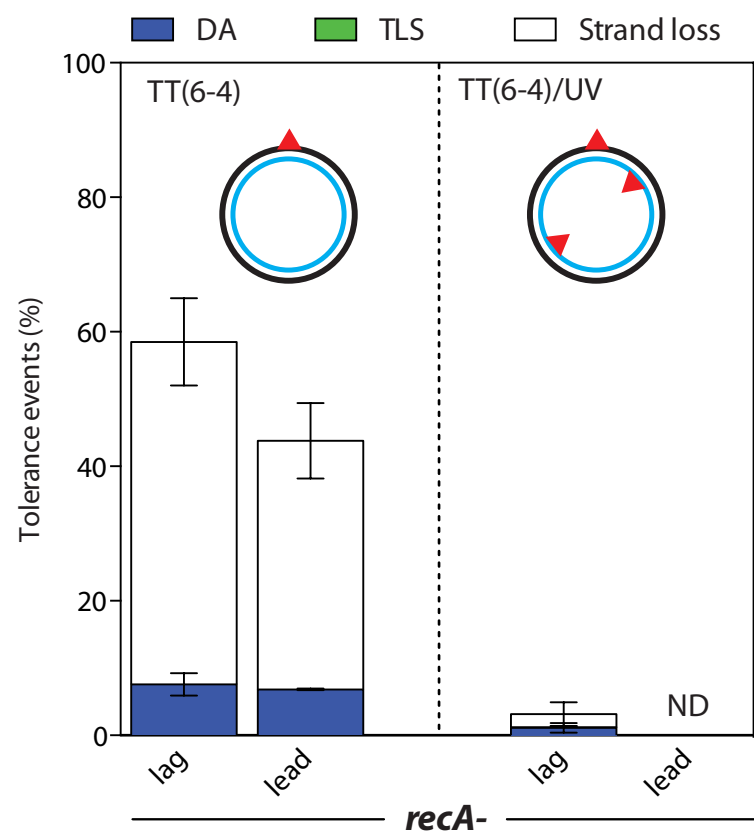

Supplement: S3 Fig — The graph represents the partition of DDT mechanisms (HDGR, TLS and damaged chromatid loss) after integration of the plasmid containing a single TT6-4 lesion (indicated as TT6-4) and a plasmid containing a single TT6-4 lesion and an average of 2–3 UV lesions in the complementary strand (indicated as TT6-4/UV) in a recA deficient strain. The TT6-4 lesion has been inserted in both orientation of the replication fort, i.e. leading (lead) and lagging (lag). Tolerance events (Y axis) represent the percentage of cells able to survive in presence of the integrated lesion compared to the lesion-free control. The data represent the average and standard deviation of at least three independent experiments. The data for the construction TT6-4/UV have been corrected taking into account the percentage of plasmids without additional UV lesions in the complementary strand (see Methods). ND = no cell survival was observed. (PDF) [file pgen.1005757.s003.pdf]

S4 Fig

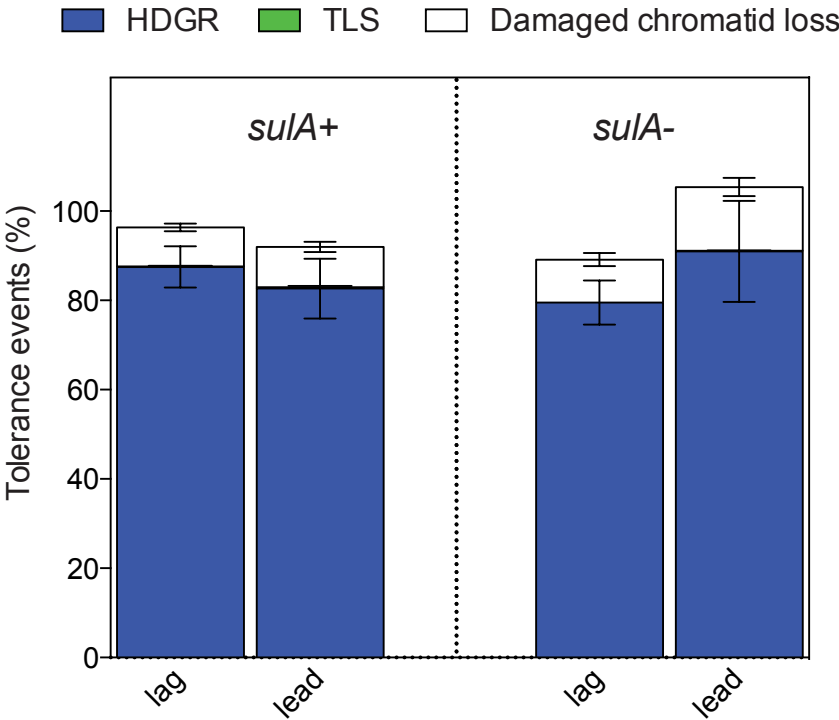

Supplement: S4 Fig — The graph represents the partition of DDT mechanisms for the UV lesion TT6-4 relative to lesion-free plasmid, in a sulA- strain. The lesion has been inserted in both orientation of the replication fork, i.e. leading (lead) and lagging (lag). Tolerance events (Y axis) represent the percentage of cells able to survive in presence of the integrated lesion compared to the lesion-free control. The data represent the average and standard deviation of at least three independent experiments. For a better comparison we included in the graph the previous results obtained in the parental strain. (PDF) [file pgen.1005757.s004.pdf]
